# Ayahuasca and Its DMT- and β-carbolines – Containing Ingredients Block the Expression of Ethanol-Induced Conditioned Place Preference in Mice: Role of the Treatment Environment

**DOI:** 10.3389/fphar.2018.00561

**Published:** 2018-05-29

**Authors:** Elisangela G. Cata-Preta, Yasmim A. Serra, Eliseu da C. Moreira-Junior, Henrique S. Reis, Natali D. Kisaki, Matheus Libarino-Santos, Raiany R. R. Silva, Thaísa Barros-Santos, Lucas C. Santos, Paulo C. R. Barbosa, José L. Costa, Alexandre J. Oliveira-Lima, Lais F. Berro, Eduardo A. V. Marinho

**Affiliations:** ^1^Department of Health Sciences, Universidade Estadual de Santa Cruz, Ilhéus, Brazil; ^2^Department of Philosophy and Human Sciences, Universidade Estadual de Santa Cruz, Ilhéus, Brazil; ^3^Faculty of Pharmaceutical Sciences, University of Campinas, Campinas, Brazil; ^4^Department of Psychiatry and Human Behavior, University of Mississippi Medical Center, Jackson, MS, United States

**Keywords:** ayahuasca, N, N-dimethyltryptamine, β-carboline alkaloids, ethanol, reward, conditioned place preference, mice

## Abstract

Ayahuasca is a hallucinogenic beverage produced from the decoction of *Banisteriopsis caapi* (Bc) and *Psychotria viridis* (Pv), β-carboline- and *N,N*-dimethyltryptamine(DMT)-containing plants, respectively. Accumulating evidence suggests that ayahuasca may have therapeutic effects on ethanol abuse. It is not known, however, whether its effects are dependent on the presence of DMT or if non-DMT-containing components would have therapeutic effects. The aim of the present study was to investigate the rewarding properties of ayahuasca (30, 100, and 300 mg/kg, orally), Bc (132, 440, and 1320 mg/kg, orally) and Pv (3.75, 12.5 and 37.5 mg/kg, i.p.) extracts and their effects on ethanol (1.8 g/kg, i.p.) reward using the conditioned place preference (CPP) paradigm in male mice. Animals were conditioned with ayahuasca, Bc or Pv extracts during 8 sessions. An intermediate, but not a high, dose of ayahuasca induced CPP in mice. Bc and Pv did not induce CPP. Subsequently, the effects of those extracts were tested on the development of ethanol-induced CPP. Ayahuasca, Bc or Pv were administered before ethanol injections during conditioning sessions. While Bc and Pv exerted no effects on ethanol-induced CPP, pretreatment with ayahuasca blocked the development of CPP to ethanol. Finally, the effects of a post-ethanol-conditioning treatment with ayahuasca, Bc or Pv on the expression of ethanol-induced CPP were tested. Animals were conditioned with ethanol, and subsequently treated with either ayahuasca, Bc or Pv in the CPP environment previously associated with saline or ethanol for 6 days. Animals were then reexposed to ethanol and ethanol-induced CPP was quantified on the following day. Treatment with all compounds in the ethanol-paired environment blocked the expression of ethanol-induced CPP. Administration of an intermediate, but not a high, dose of ayahuasca and Bc, as well as Pv administration, in the saline-paired compartment blocked the expression of ethanol-induced CPP. The present study sheds light into the components underlying the therapeutic effects of ayahuasca on ethanol abuse, indicating that ayahuasca and its plant components can decrease ethanol reward at doses that do not exert abuse liability. Importantly, the treatment environment seems to influence the therapeutic effects of ayahuasca and Bc, providing important insights into clinical practice.

## Introduction

Alcohol (ethanol, Eth) use disorder (AUD) and its health consequences are a major public health problem. The World Health Organization estimated that the global prevalence of AUD in 2010 was 4.1%, including Eth dependence (2.3%) and harmful use of Eth (1.8%) (World Health Organization [WHO], 2014). Eth use is the third leading risk factor for poor health globally, and it is estimated that nearly 2.5 million deaths every year are attributable to Eth use (World Health Organization [WHO], 2010). Thus, despite the substantial amount of resources that governments and international organizations invest in programs to prevent substance use disorders, AUD is still high across the globe. Currently available treatments are only partially effective (Mason, 2017) and further research on new treatment approaches is needed.

Throughout the last two decades, accumulating evidence has suggested that ayahuasca (Aya) may have therapeutic properties on substance use disorders (for review, see Nunes et al., 2016). Aya is a brew frequently prepared by the decoction of *Banisteriopsis caapi* and *Psychotria viridis*, β-carboline- and *N,N*-dimethyltryptamine(DMT)-containing plants, respectively. Originally used for religious purposes by Amerindian populations of the Amazon Basin, the use of Aya has spread throughout the world (Tupper, 2008), being currently used in syncretic religions such as Santo Daime and União do Vegetal and other contexts, such as Aya retreats (Labate et al., 2009; Goulart, 2011; Luna, 2011). Case-control studies have found a lower prevalence of substance use and substance-related problems in Aya users from União do Vegetal and Santo Daime religions relative to control groups (Grob et al., 1996; Da Silveira et al., 2005; Doering-Silveira et al., 2005; Fabregas et al., 2010; Barbosa et al., 2016), and decreased substance use after joining those churches (Halpern et al., 2008; Labate et al., 2014). In addition, studies have shown that Aya-assisted therapy resulted in decreased drug use and craving in drug dependent individuals (Thomas et al., 2013; Loizaga-Velder and Verres, 2014).

Although increasing evidence supports the effectiveness of Aya for the treatment of drug abuse, it remains unknown whether Aya has anti-addictive properties alone or if other factors, such as contextual and religious influences, play a major role in the results described above. In what seems to be the only study to date investigating the effects of Aya on an animal model of drug abuse, a previous study from our group investigated the effects of Aya on behaviors induces by acute and chronic Eth administration in mice (Oliveira-Lima et al., 2015). Aya blocked the development and expression of acute and chronic Eth-induced hyperlocomotion and behavioral sensitization without affecting baseline locomotor activity (Oliveira-Lima et al., 2015). Those findings suggest that Aya may have therapeutic effects *per se* that are not completely dependent on sociocultural religious variables.

Importantly, it is well known that DMT has no significant psychoactive effect when ingested orally due to its breakdown by monoamine oxidase A (MAO-A) in the gastrointestinal tract (Lanaro et al., 2015). According to the proposed pharmacokinetic model of Aya, β-carboline alkaloids present in *B. caapi* inhibit MAO-A, thereby allowing the entry of DMT from *P. viridis* to systemic circulation and central nervous system, and its subsequent psychoactive effects (Holmstedt and Lindgren, 1967; McKenna, 2004). Thus, it was believed that the main psychoactive effects of Aya were attributable to DMT, with β-carboline alkaloids such as harmine and harmaline simply facilitating its effects. However, more recent evidence suggests that β-carboline alkaloids also have psychoactive properties and could exert therapeutic effects in drug abuse (for review, see Brierley and Davidson, 2012). Studies have shown a decrease in morphine and cocaine intake after harmaline administration (Glick et al., 1994), as well as harmine-induced attenuation of morphine withdrawal (Aricioglu-Kartal et al., 2003). Therefore, it remains unknown whether the effects of Aya are dependent on the presence of DMT or if non-DMT containing components, such as the plant *Banisteriopsis caapi*, would have therapeutic effects *per se*.

The aim of the present study was to investigate the rewarding properties of Aya (prepared by the decoction of the stems of the *Banisteriopsis caapi* vine combined with the leaves of the *Psychotria viridis* bush), *Banisteriopsis caapi* and *Psychotria viridis* extracts. We also investigated the effects of those compounds on the rewarding properties of Eth using the conditioned place preference (CPP) paradigm (Liu et al., 2008). We evaluated the effects of Aya, *Banisteriopsis caapi* or *Psychotria viridis* extracts alone on CPP as well as on the development of CPP to Eth. Additionally, we also investigated if the treatment environment could influence the effects of those extracts on the subsequent expression of Eth-induced CPP. Thus, we also tested the effects of treatments with Aya, *Banisteriopsis caapi* or *Psychotria viridis* extracts in the CPP environment previously associated with saline (unpaired) or Eth (paired) on the subsequent expression of CPP to Eth.

## Materials and Methods

### Animals

Three-month-old Swiss male mice from our own colony were used. Animals weighing 35–40 g were group housed (5–7 per cage) in polypropylene cages (32 × 42 × 18 cm) under controlled temperature (22–23°C) and light (12 h light, 12 h dark; lights on at 6 h 45) conditions. Rodent chow (Nuvilab, Quimtia SA, Colombo, PR, Brazil) and water were available *ad libitum* throughout the experiments. Animals were maintained according to the National Institutes of Health Guide for the Care and Use of Laboratory Animals (8th Edition, revised 2011) and in accordance with the Brazilian Law for Procedures for Animal Scientific Use (#11794/2008). The Institutional Animal Care and Use Committee of UESC approved the experimental procedures.

### Preparation of Extracts and Compounds Analysis

A batch of Aya had been previously obtained from a member of the Santo Daime church, lyophilized and analyzed for the quantification of the amount DMT, tetrahydroharmine, harmine and harmaline, as previously described (Oliveira-Lima et al., 2015). Samples of *B. caapi* and *P. viridis* were obtained from members of the Santo Daime church and submitted to extraction. The extracts were, then, lyophilized, rendering a freeze dried material that was analyzed as described below. The ratio of dry extract/volume of liquid (extract) was calculated to establish the doses to be administered in the experiments for all extracts.

In order to quantify the amount of the main compounds of *B. caapi* (tetrahydroharmine, harmine, and harmaline) and *P. viridis* (DMT) in our preparation, the extracts were analyzed by liquid chromatography mass spectrometry (LC-MS/MS) conducted on a high performance liquid chromatography equipment Prominence system (Shimadzu, Kyoto, Japan). Harmine hydrochloride and harmaline hydrochloride were purchased from Sigma^®^. The synthesis of tetrahydroharmine was performed as previously described (Callaway et al., 1996). DMT was synthesized according to a modified procedure based on the selective dimethylation method (Giumanini and Casalini, 1980; Pires et al., 2009). The stock solutions (1.0 mg/ml) of harmine, harmaline, tetrahydroharmine and DMT were prepared in methanol and stored at -20°C until the performance of the LC-MS/MS.

### Drugs

Eth (Merck^®^), Aya, extract of *B. caapi* (EBc) and extract of *P. viridis* (EPv) were dissolved in 0.9% saline (Sal) solution, which was used as vehicle (Veh) solution for the Aya, EBc and EPv treatments. Eth and Sal solutions were administered intraperitoneally (i.p.) at 10 ml/kg of body weight. Aya, EBc and Veh were orally administered by gavage at a volume of 10 ml/kg. Because DMT has no significant psychoactive effect when ingested orally due to its breakdown by monoamine oxidase A (MAO-A) in the gastrointestinal tract (Lanaro et al., 2015), oral administration of DMT would not allow us to evaluate the effects of this compound in the behavioral tests. Thus, EPv and its Veh were administered i.p. at 10 ml/kg of body weight. The selected dose range of Aya and Eth was based on previous studies from our group (Marinho et al., 2015; Oliveira-Lima et al., 2015; Silva et al., 2017). Doses of Aya were chosen based on their ability to prevent acute Eth-induced hyperlocomotion and locomotor sensitization in mice (Oliveira-Lima et al., 2015). For comparative purposes, the doses of EBc and EPv were calculated based on the concentration of harmine and DMT, respectively, in the Aya extract used in the present study, as previously described (Oliveira-Lima et al., 2015).

### Conditioned Place Preference

The CPP apparatus consisted of 2 conditioning compartments of equal size (40 × 20 × 20 cm): 1 black with white vertical bands in the walls and a black wooden floor and 1 white with black horizontal bands in the walls and a green (red) smooth floor, both connected by a central choice compartment (40 × 10 × 15 cm) that was accessible by sliding doors. The CPP procedure consisted of the following phases: Experiments 1, 2, 3, and 4 – habituation, pre-conditioning test, conditioning, post-conditioning test; Experiments 5, 6, 7, and 8 – habituation, pre-conditioning test, conditioning, post-conditioning test, treatment, Eth reexposure and post-treatment test.

#### Habituation and Pre-conditioning Test

In order to avoid a novelty effect and establish if animals showed a preference for either of the compartments, a habituation session and a pre-conditioning test were conducted (Days 1 and 2, respectively) in which animals were placed in the center of the apparatus with the door open with free access to each compartment for 15 min. No injection was administered on the habituation day or on the day of the pre-conditioning test.

#### Conditioning

An unbiased design was used because mice showed no preference for either of the compartments in the pre-conditioning test. Therefore, animals were randomly assigned to an experimental group and a ‘drug-paired compartment’ in a counterbalanced fashion, with the “black” compartment as the drug-paired one for half of the animals and the “white” compartment for the other half. One “drug-paired compartment” and one “Sal-paired compartment” were defined for all animals. The conditioning trials were performed during 8 consecutive days (Days 3–10). During the conditioning sessions, the doors remained closed so the animals would be confined to one of the conditioning compartments. Animals received Sal on even days. On odd days, animals received Aya (30, 100, and 300 mg/kg, Experiment 1a), EBc (132, 440, and 1320 mg/kg, Experiment 1b), EPv (3.75, 12.5, and 37.5 mg/kg, Experiment 1c) or Eth (1.8 g/kg, Experiments 2a,b,c, 3a,b, 4a,b, and 5a,b). Five (Eth), 30 (Aya, EBc) or 20 (EPv) min after treatments, mice were confined to the assigned drug- or Sal-paired compartment for 10 min. On Experiments 2a, 2b, and 2c, animals received Veh, Aya (100 or 300 mg/kg), EBc (440 or 1320 mg/kg) or EPv (12.5 or 37.5 mg/kg) 25 (Aya, EBc) or 15 (EPv) min before Eth injections.

#### Post-conditioning Test

Twenty four hours after the last conditioning session (Day 11), animals were placed in the center of the apparatus with the door open with free access to each compartment for 15 min. No injection was administered on the day of the post-conditioning test.

#### Treatment

Treatment for 8 consecutive days (Days 12 to 19), animals received every other day administrations of Veh, Aya (100 and 300 mg/kg, Experiments 3a and 3b), EBc (440 and 1320 mg/kg, Experiments 4a and 4b) or EPv (12.5 or 37.5 mg/kg, Experiments 5a and 5b) and, 30 (Aya, EBc) or 20 (EPv) min after treatments, were confined to the assigned Eth- or Sal-paired compartment for 10 min.

#### Ethanol Reexposure

Twenty four hours after the last treatment session (Day 20), animals received an injection of Eth (1.8 g/kg) and, five min after injection, were confined to the Eth-paired compartment for 10 min.

#### Post-treatment Test

Twenty four hours after the Eth reexposure session (Day 21), animals were placed in the center of the apparatus with the door open with free access to each compartment for 15 min. No injection was administered on the day of the post-treatment test.

**FIGURE 1:**
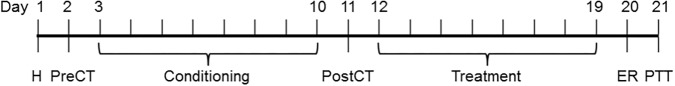
Conditioned place preference (CPP) protocol design. H, habituation; PreCT, drug-free pre-conditioning test; Conditioning, ayahuasca (30, 100, or 300 mg/kg, Experiment 1), *Banisteriopsis caapi* (132, 440 or 1320 mg/kg, Experiment 2), ethanol (1.8 g/kg, Experiments 5, 6, 7 and 8) – preceded by vehicle or ayahuasca (100 or 300 mg/kg, Experiment 3) or *Banisteriopsis caapi* (440 or 1320 mg/kg, Experiment 4) pretreatment – or saline conditioning; PostCT, drug-free post-conditioning test; Treatment, vehicle, ayahuasca (100 or 300 mg/kg, Experiments 5 and 6) or *Banisteriopsis caapi* (440 or 1320 mg/kg, Experiments 7 and 8) treatment in the compartment previously paired with ethanol (Experiments 5 and 7) or saline (Experiments 6 and 8); ER, ethanol (1.8 g/kg) reexposure in previously ethanol-paired compartment; PTT, drug-free post-treatment test.

All behavioral sessions started between 8 and 9 am for all experiments in order to minimize the effect of time of the day and circadian rhythms on the behavioral tasks being conducted. Because several groups were ran concomitantly within a given experiment, the order of animals being submitted to the behavioral sessions in the CPP was randomized for each phase described above, so that all groups had animals being tested at the same time. The CPP apparatus was cleaned with Eth-water (5%) solution before each behavioral session/test to eliminate possible bias due to odors left by previous mice. During test sessions, the time spent in each compartment was registered. Movement was tracked using the ANY-maze software (version 5.1, Stoelting) and a webcam suspended overhead. Expression of drug-induced CPP was evidenced by the CPP score (difference between the time spent in the drug-paired and in the non-drug-paired compartments). The CPP protocol design is illustrated in **Figure 1**. Different cohorts of mice were used for each experiment (as well as sub-experiment) described below.

### Experimental Design

#### Experiment 1: Effects of Treatment With Ayahuasca, *Banisteriopsis caapi* or *Psychotria viridis* on the CPP Paradigm

In order to evaluate if Aya, EBc or EPv would induce CPP, mice were submitted to the habituation, pre-conditioning test and Aya (*Experiment 1a*, *n* = 8 per group), EBc (*Experiment 1b*, *n* = 8 per group) or EPv (*Experiment 1c*, *n* = 8 per group) conditioning followed by post-conditioning test as previously described.

#### Experiment 2: Effects of Pretreatment With Ayahuasca, *Banisteriopsis caapi* or *Psychotria viridis* on the Development of Ethanol-Induced CPP

Mice were submitted to the habituation, pre-conditioning test, Eth conditioning preceded by Aya (*Experiment 2a*, *n* = 8 per group), EBc (*Experiment 2b*, *n* = 8 per group) or EPv (*Experiment 2c*, *n* = 8 per group) pretreatments and post-conditioning test as previously described.

#### Experiment 3: Role of the Treatment Environment in the Effects of Ayahuasca on the Expression of Ethanol-Induced CPP

Mice were submitted to the pre-conditioning test, Eth conditioning and post-conditioning test as previously described. Twenty-four hours after the post-conditioning test, the treatment phase began. For 8 days, animals received an oral administration of either Veh or Aya every other day on even days and, 30 min after injection, were confined to the compartment previously paired with Eth (*Experiment 3a*, *n* = 8 per group) or Sal (*Experiment 3b*, *n* = 8 per group) for 10 min. On odd days, animals received an oral administration of Veh associated with the opposite (Eth- or Sal-paired) compartment. The treatment phase was followed by the Eth reexposure and drug-free post-treatment test sessions as previously described.

#### Experiment 4: Role of the Treatment Environment in the Effects of *Banisteriopsis caapi* on the Expression of Ethanol-Induced CPP

Mice were submitted to the pre-conditioning test, Eth conditioning and post-conditioning test as previously described. Twenty-four hours after the post-conditioning test, the treatment phase began. For 8 days, animals received an oral administration of either Veh or EBc every other day on even days and, 30 min after injection, were confined to the compartment previously paired with Eth (*Experiment 4a*, *n* = 8 per group) or Sal (*Experiment 4b*, *n* = 8 per group) for 10 min. On odd days, animals received an oral administration of Veh associated with the opposite (Eth- or Sal-paired) compartment. The treatment phase was followed by the Eth reexposure and drug-free post-treatment test sessions as previously described.

#### Experiment 5: Role of the Treatment Environment in the Effects of *Psychotria viridis* on the Expression of Ethanol-Induced CPP

Mice were submitted to the pre-conditioning test, Eth conditioning and post-conditioning test as previously described. Twenty-four hours after the post-conditioning test, the treatment phase began. For 8 days, animals received an i.p. administration of either Veh or EPv every other day on even days and, 20 min after injection, were confined to the compartment previously paired with Eth (*Experiment 5a*, *n* = 8 per group) or Sal (*Experiment 5b*, *n* = 8 per group) for 10 min. On odd days, animals received an i.p. administration of Veh associated with the opposite (Eth- or Sal-paired) compartment. The treatment phase was followed by the Eth reexposure and drug-free post-treatment test sessions as previously described.

### Statistical Analysis

All variables were checked for normality (Shapiro–Wilk test) and homogeneity of variances (Levene’s test), which validated the use of the parametric test. Within-group comparisons were performed using the paired samples *t*-test. CPP score was analyzed using the Student’s *t*-test. Multiple comparisons were performed using one- or two-way analysis of variance (ANOVA), with repeated measures (RM) or not, and Bonferroni *post hoc* test when necessary. A probability of *p* < 0.05 was considered a statistically significant difference.

## Results

### *Banisteriopsis caapi* Compounds Analysis

LC-MS/MS analysis indicated the following active constituents in our EBc sample:

– Tetrahydroharmine: < 0.1 mg/100 mg
– Harmine: 0.876 mg/100 mg
– Harmaline: 0.927 mg/100 mg

### *Psychotria viridis* Compounds Analysis

LC-MS/MS analysis indicated the following active constituent in our EPv sample:

– *N,N*-dimethyltryptamine (DMT): 3.2 mg/100 mg

### Experiment 1: Effects of Treatment With Ayahuasca, *Banisteriopsis caapi* or *Psychotria viridis* on the CPP Paradigm

#### Experiment 1a (Ayahuasca)

**FIGURE 2:**
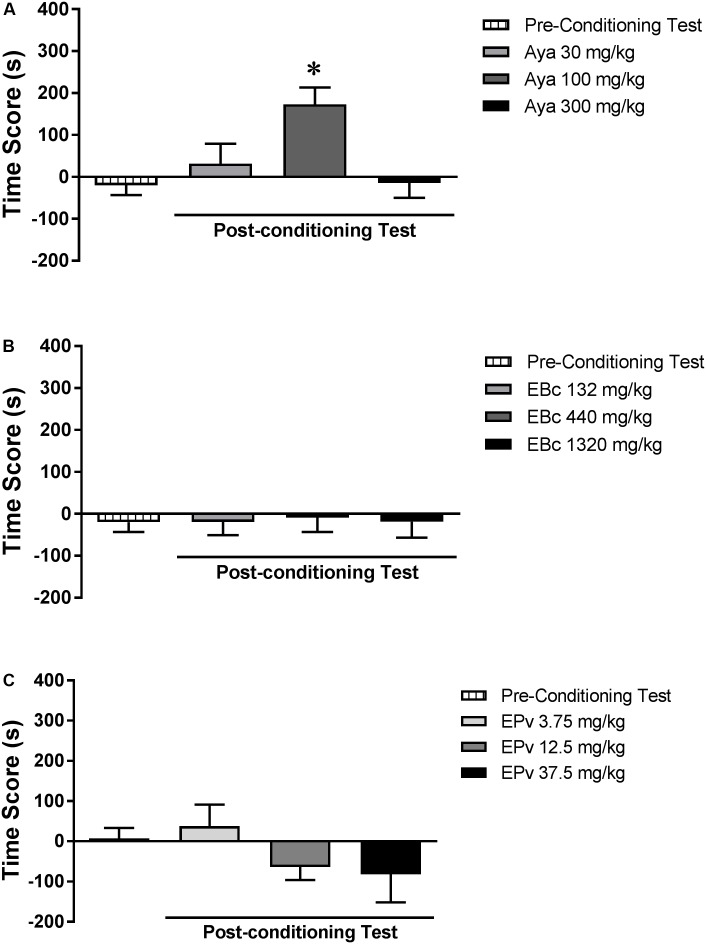
Effects of **(A)** ayahuasca (Aya), **(B)** extract of *Banisteriopsis caapi* (EBc) and **(C)** extract of *Psychotria viridis* (EPv) on the conditioned place preference (CPP) paradigm. CPP score (difference between the time spent in the drug-paired and in the saline-paired compartments) during the pre-conditioning and post-conditioning test sessions. Data are reported as means ± SEM. ^∗^*p* < 0.05 compared with the pre-conditioning test.

Analysis of the pre-conditioning test showed that animals had no preference for the subsequently Sal- or Aya-paired compartments [*t*(23) = 0.89; *p* > 0.05] (data not shown). One-way ANOVA showed no significant differences between groups (Aya 30, Aya 100, or Aya 300) during the pre-conditioning test [*F*(2,21) = 1.20, *p* > 0.05] (data not shown). Two-way RM ANOVA showed a significant interaction effect between time (pre- vs. post-conditioning) and Aya dose [*F*(2,21) = 5.99, *p* < 0.01] (**Figure 2A**). Bonferroni *post hoc* test showed that animals treated with 100 mg/kg, but not 30 or 300 mg/kg, Aya showed higher levels of CPP score in the post-conditioning test compared to the pre-conditioning test, indicating a marked preference for the 100 mg/kg Aya-paired compartment.

#### Experiment 1b (*Banisteriopsis caapi*)

Analysis of the pre-conditioning test showed that animals had no preference for the subsequently Sal- or EBc-paired compartments [*T*(23) = 0.86; *p* > 0.05] (data not shown). One-way ANOVA showed no significant differences between groups (EBc 132, EBc 440, or EBc 1320) during the pre-conditioning test [*F*(2,21) = 1.12, *p* > 0.05] (data not shown). Two-way RM ANOVA showed no significant effect of time (pre- vs. post-conditioning) [*F*(1,21) = 0.01, *p* > 0.05], EBc dose [*F*(2,21) = 0.55, *p* > 0.05] or interaction between the two factors [*F*(2,21) = 0.64, *p* > 0.05] (**Figure 2B**).

#### Experiment 1c (*Psychotria viridis*)

Analysis of the pre-conditioning test showed that animals had no preference for the subsequently Sal- or EPv-paired compartments [*T*(23) = 0.21; *p* > 0.05] (data not shown). One-way ANOVA showed no significant differences between groups (EPv 3.75, EPv 12.5, or EPv 37.5) during the pre-conditioning test [*F*(2,21) = 0.78, *p* > 0.05] (data not shown). Two-way RM ANOVA showed no significant effect of time (pre- vs. post-conditioning) [*F*(1,21) = 0.74, *p* > 0.05] or interaction between time and EPv dose [*F*(2,21) = 0.24, *p* > 0.05], but showed a significant effect of EPv dose alone [*F*(2,21) = 3.77, *p* < 0.05] (**Figure 2C**). Bonferroni *post hoc* test, however, showed no differences in the CPP scores of groups during the post-conditioning session compared to the pre-conditioning session.

### Experiment 2: Effects of Pretreatment With Ayahuasca, *Banisteriopsis caapi* or *Psychotria viridis* on the Development of Ethanol-Induced CPP

#### Experiment 2a (Ayahuasca)

**FIGURE 3:**
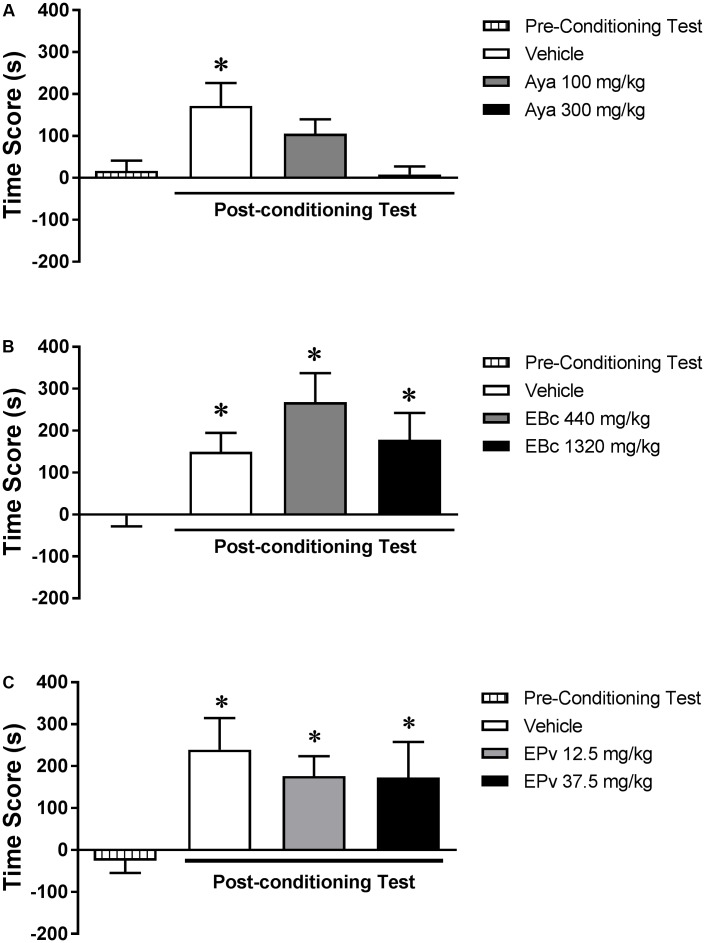
Effects of pretreatments with **(A)** ayahuasca (Aya), **(B)** extract of *Banisteriopsis caapi* (EBc) or **(C)** extract of *Psychotria viridis* (EPv) before ethanol conditioning sessions on the development of ethanol-induced conditioned place preference (CPP). CPP score (difference between the time spent in the ethanol-paired and in the saline-paired compartments) during the pre-conditioning and post-conditioning test sessions. Data are reported as means ± SEM. ^∗^*p* < 0.05 compared with the pre-conditioning test.

Analysis of the pre-conditioning test showed that animals had no preference for the subsequently Sal- or Eth-paired compartments [*T*(23) = 0.67; *p* > 0.05] (data not shown). One-way ANOVA showed no significant differences between groups (Vehicle, Aya 100 or Aya 300) during the pre-conditioning test [*F*(2,21) = 2.12, *p* > 0.05] (data not shown). Two-way RM ANOVA showed a significant interaction effect between time (pre- vs. post-conditioning) and Aya treatment [*F*(2,21) = 3.12, *p* < 0.05] (**Figure 3A**). Bonferroni *post hoc* test showed that animals treated with Veh before Eth administrations showed higher levels of CPP score in the post-conditioning test compared to the pre-conditioning test, indicating a marked preference for the Eth-paired compartment. This effect was not observed for animals treated with 100 mg/kg and 300 mg/kg Aya before Eth conditioning sessions, indicating that the development of Eth-induced CPP was blocked by co-administration of Aya.

#### Experiment 2b (*Banisteriopsis caapi*)

Analysis of the pre-conditioning test showed that animals had no preference for the subsequently Sal- or Eth-paired compartments [*T*(23) = 0.1; *p* > 0.05] (data not shown). One-way ANOVA showed no significant differences between groups (Vehicle, EBc 440 or EBc 1320) during the pre-conditioning test [*F*(2,21) = 0.92, *p* > 0.05] (data not shown). Two-way RM ANOVA showed a significant effect of time (pre- vs. post-conditioning) [*F*(1,21) = 26.18, *p* < 0.0001], but not EBc treatment [*F*(2,21) = 0.74, *p* > 0.05] or interaction between the two factors [*F*(2,21) = 2.00, *p* > 0.05] (**Figure 3B**). Bonferroni *post hoc* test showed that animals treated with Veh before Eth administrations showed higher levels of CPP score in the post-conditioning test compared to the pre-conditioning test, indicating a marked preference for the Eth-paired compartment, an effect that was not affected by treatment with EBc.

#### Experiment 2c (*Psychotria viridis*)

Analysis of the pre-conditioning test showed that animals had no preference for the subsequently Sal- or Eth-paired compartments [*T*(23) = 0.43; *p* > 0.05] (data not shown). One-way ANOVA showed no significant differences between groups (Vehicle, EPv 12.5 or EPv 37.5) during the pre-conditioning test [*F*(2,21) = 0.00, *p* > 0.05] (data not shown). Two-way RM ANOVA showed a significant effect of time (pre- vs. post-conditioning) [*F*(1,21) = 21.15, *p* < 0.001], but not EPv treatment [*F*(2,21) = 0.86, *p* > 0.05] or interaction between the two factors [*F*(2,21) = 0.21, *p* > 0.05] (**Figure 3C**). Bonferroni *post hoc* test showed that animals treated with Veh before Eth administrations showed higher levels of CPP score in the post-conditioning test compared to the pre-conditioning test, indicating a marked preference for the Eth-paired compartment, an effect that was not affected by treatment with EPv.

### Experiment 3: Role of the Treatment Environment in the Effects of Ayahuasca on the Expression of Ethanol-Induced CPP

#### Experiment 3a (Ethanol-Paired Compartment)

**FIGURE 4:**
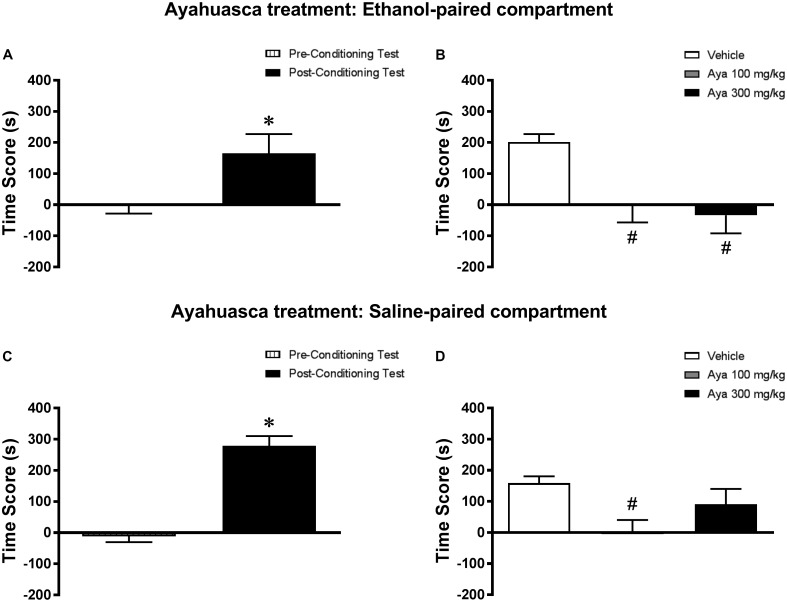
Effects of post-conditioning treatments with ayahuasca on the expression ethanol-induced conditioned place preference (CPP). **(A,C)** CPP score (difference between the time spent in the ethanol-paired and in the saline-paired compartments) during the pre-conditioning test and the post-ethanol conditioning test sessions. **(B,D)** CPP score during the post-treatment test conducted after treatment with ayahuasca (Aya) in the **(B)** ethanol- or **(D)** saline-paired compartments and subsequent ethanol reexposure. Data are reported as means ± SEM. ^∗^*p* < 0.05 compared with the Pre-Conditioning Test; ^#^*p* < 0.05 compared with the Vehicle group.

Analysis of the pre-conditioning test showed that animals had no preference for the subsequently Sal- or Eth-paired compartments [*T*(23) = 0.53; *p* > 0.05] (data not shown). Paired samples *t*-test showed that animals displayed a significant increase in CPP score during the post-conditioning test compared to the pre-conditioning test [*T*(23) = 4.32; *p* < 0.001] (**Figure 4A**). In the post-treatment test, one-way ANOVA followed by Bonferroni *post hoc* test showed that animals treated with 100 and 300 mg/kg Aya displayed a significant decrease in CPP score during the post-treatment test compared to Veh-treated animals [*F*(2,21) = 7.31, *p* < 0.01] (**Figure 4B**), indicating that the expression of Eth-induced CPP was blocked by treatment with Aya in the Eth-paired compartment.

#### Experiment 3b (Saline-Paired Compartment)

Analysis of the pre-conditioning test showed that animals had no preference for the subsequently Sal- or Eth-paired compartments [*T*(23) = 0.63; *p* > 0.05] (data not shown). Paired samples t-test showed that animals displayed a significant increase in CPP score during the post-conditioning test compared to the pre-conditioning test [*T*(23) = 8.38; *p* < 0.0001] (**Figure 4C**). In the post-treatment test, one-way ANOVA followed by Bonferroni *post hoc* test showed that animals treated with 100 mg/kg, but not 300 mg/kg, Aya displayed a significant decrease in CPP score during the post-treatment test compared to Veh-treated animals [*F*(2,21) = 2.3, *p* < 0.05] (**Figure 4D**), indicating that the expression of Eth-induced CPP was blocked by treatment with 100 mg/kg Aya in the Sal-paired compartment.

#### Environment vs. Treatment Interaction

When performing a combined analysis of the results for Experiments 3a and 3b, two-way ANOVA showed a significant interaction effect between treatment environment (Eth- vs. Sal-paired compartment) and Aya treatment [*F*(2,42) = 4.15, *p* < 0.05]. Bonferroni *post hoc* test showed that animals treated with 300 mg/kg Aya in the Eth-paired environment showed lower levels of CPP score during the post-treatment test compared to animals treated with 300 mg/kg Aya in the Sal-paired environment. No statistically significant differences were observed for groups treated with Veh and 100 mg/kg Aya.

### Experiment 4: Role of the Treatment Environment in the Effects of *Banisteriopsis caapi* on the Expression of Ethanol-Induced CPP

#### Experiment 4a (Ethanol-Paired Compartment)

**FIGURE 5:**
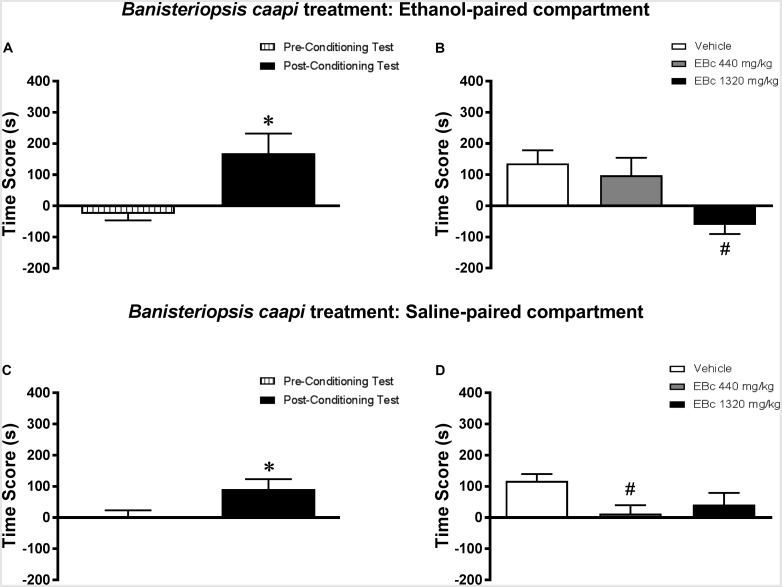
Effects of post-conditioning treatments with *Banisteriopsis caapi* on the expression ethanol-induced conditioned place preference (CPP). **(A,C)** CPP score (difference between the time spent in the ethanol-paired and in the saline-paired compartments) during the pre-conditioning test and the post-ethanol conditioning test sessions. **(B,D)** CPP score during the post-treatment test conducted after treatment with extract of *Banisteriopsis caapi* (EBc) in the **(B)** ethanol- or **(D)** saline-paired compartments and subsequent ethanol reexposure. Data are reported as means ± SEM. ^∗^*p* < 0.05 compared with the Pre-Conditioning Test; ^#^*p* < 0.05 compared with the Vehicle group.

Analysis of the pre-conditioning test showed that animals had no preference for the subsequently Sal- or Eth-paired compartments [*T*(23) = 1.19; *p* > 0.05] (data not shown). Paired samples *t*-test showed that animals displayed a significant increase in CPP score during the post-conditioning test compared to the pre-conditioning test [*T*(23) = 2.91; *p* < 0.01] (**Figure 5A**). In the post-treatment test, one-way ANOVA followed by Bonferroni *post hoc* test showed that animals treated with 1320 mg/kg, but not 440 mg/kg, EBc displayed a significant decrease in CPP score during the post-treatment test compared to Veh-treated animals [*F*(2,21) = 5.72, *p* < 0.05] (**Figure 5B**), indicating that the expression of Eth-induced CPP was blocked by treatment with 1320 mg/kg EBc in the Eth-paired compartment.

#### Experiment 4b (Saline-Paired Compartment)

Analysis of the pre-conditioning test showed that animals had no preference for the subsequently Sal- or Eth-paired compartments [*T*(23) = 0.01; *p* > 0.05] (data not shown). Paired samples t-test showed that animals displayed a significant increase in CPP score during the post-conditioning test compared to the pre-conditioning test [*T*(23) = 2.69; *p* < 0.05] (**Figure 5C**). In the post-treatment test, one-way ANOVA followed by Bonferroni *post hoc* test showed that showed that animals treated with 440 mg/kg, but not 1320 mg/kg, EBc displayed a significant decrease in CPP score during the post-treatment test compared to Veh-treated animals [*F*(2,29) = 5.86, *p* < 0.01] (**Figure 5D**), indicating that the expression of Eth-induced CPP was blocked by treatment with 440 mg/kg EBc in the Sal-paired compartment.

#### Environment vs. Treatment Interaction

When performing a combined analysis of the results for Experiments 4a and 4b, two-way ANOVA showed a significant interaction effect between treatment environment (Eth- vs. Sal-paired compartment) and EBc treatment [*F*(2,42) = 3.53, *p* < 0.05]. Bonferroni *post hoc* test showed no statistically significant differences between scores obtained in the Eth- vs. Sal-paired compartments for groups treated with Veh, 440 or 1320 mg/kg EBc.

### Experiment 5: Role of the Treatment Environment in the Effects of *Psychotria viridis* on the Expression of Ethanol-Induced CPP

#### Experiment 5a (Ethanol-Paired Compartment)

**FIGURE 6:**
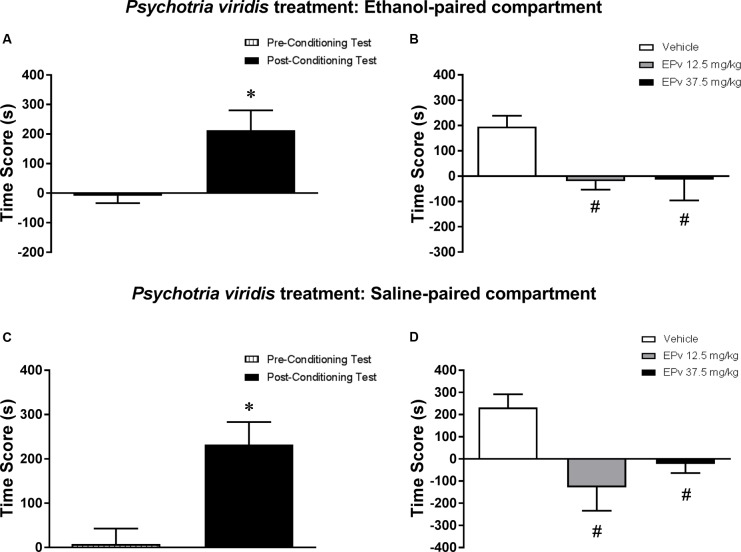
Effects of post-conditioning treatments with and *Psychotria viridis* on the expression ethanol-induced conditioned place preference (CPP). **(A,C)** CPP score (difference between the time spent in the ethanol-paired and in the saline-paired compartments) during the pre-conditioning test and the post-ethanol conditioning test sessions. **(B,D)** CPP score during the post-treatment test conducted after treatment with extract of *Psychotria viridis* (EPv) in the **(B)** ethanol- or **(D)** saline-paired compartments and subsequent ethanol reexposure. Data are reported as means ± SEM. ^∗^*p* < 0.05 compared with the Pre-Conditioning Test; ^#^*p* < 0.05 compared with the Vehicle group.

Analysis of the pre-conditioning test showed that animals had no preference for the subsequently Sal- or Eth-paired compartments [*T*(23) = 0.36; *p* > 0.05] (data not shown). Paired samples *t*-test showed that animals displayed a significant increase in CPP score during the post-conditioning test compared to the pre-conditioning test [*T*(23) = 2.86; *p* < 0.01] (**Figure 6A**). In the post-treatment test, one-way ANOVA followed by Bonferroni *post hoc* test showed that animals treated with 12.5 and 37.5 mg/kg EPv displayed a significant decrease in CPP score during the post-treatment test compared to Veh-treated animals [*F*(2,21) = 4.55, *p* < 0.05] (**Figure 6B**), indicating that the expression of Eth-induced CPP was blocked by treatment with EPv in the Eth-paired compartment.

#### Experiment 5b (Saline-Paired Compartment)

Analysis of the pre-conditioning test showed that animals had no preference for the subsequently Sal- or Eth-paired compartments [*T*(23) = 0.22; *p* > 0.05] (data not shown). Paired samples t-test showed that animals displayed a significant increase in CPP score during the post-conditioning test compared to the pre-conditioning test [*T*(23) = 3.59; *p* < 0.01] (**Figure 6C**). In the post-treatment test, one-way ANOVA followed by Bonferroni *post hoc* test showed that animals treated with 12.5 and 37.5 mg/kg EPv displayed a significant decrease in CPP score during the post-treatment test compared to Veh-treated animals [*F*(2,21) = 6.3, *p* < 0.01] (**Figure 6D**), indicating that the expression of Eth-induced CPP was blocked by treatment with EPv in the Sal-paired compartment.

#### Environment vs. Treatment Interaction

When performing a combined analysis of the results for Experiments 5a and 5b, two-way ANOVA showed a significant effect of EPv treatment [*F*(2,42) = 10.66, *p* < 0.001], but not treatment environment (Eth- vs. Sal-paired compartment) [*F*(1,42) = 0.24, *p* > 0.05] or interaction between the two factors [*F*(2,42) = 0.63, *p* > 0.05]. Bonferroni *post hoc* test showed no statistically significant differences between scores obtained in the Eth- vs. Sal-paired compartments for groups treated with Veh, 12.5 or 37.5 mg/kg EPv.

## Discussion

In the present study we investigated the rewarding properties of Aya and its plant components, *Banisteriopsis caapi* (EBc) and *Psychotria viridis* (EPv), and their effects on Eth reward using the CPP paradigm. Our findings show that an intermediate, but not a high, dose of Aya exerted rewarding effects, inducing CPP in mice. Pretreatment with Aya also blocked the development of Eth-induced CPP at all doses tested. Both EBc and EPv had no effects on CPP alone or on Eth-induced CPP. Treatment with Aya, EBc and EPv in the environment previously paired to Eth blocked the subsequent expression of CPP induced by an Eth reexposure. Regarding treatments in the Sal-paired compartment, administration of an intermediate, but not a high, dose of Aya and EBc, as well as administration of EPv at both doses tested, blocked the subsequent expression of Eth-induced CPP. Statistical analysis of our findings showed a significant interaction effect between treatment environment (Eth- vs. Sal-paired compartment) and Aya or EBc, but not EPv, treatments, suggesting that the treatment environment seems to play an important role in the effects of Aya and EBc on Eth reward.

Compelling evidence indicates that the effects of Aya are mediated by serotonin 5-HT_2A_ and 5-HT_2C_ receptors. Firstly, DMT is a 5-HT_2A/2C_ receptor agonist, which has been proposed to mediate its psychoactive properties (Glennon et al., 2000). Secondly, tetrahydroharmine is a serotonin reuptake inhibitor (Buckholtz and Boggan, 1977), and harmine binds to serotonin 5-HT_2A/2C_ receptors (Grella et al., 1998; Glennon et al., 2000). The effects of harmine at 5-HT_2A/2C_ receptors have been proposed to be an agonist mechanism (Brierley and Davidson, 2013); however, a 5-HT_2_ receptor agonist or antagonist action is yet to be confirmed. Importantly, 5-HT_2A_ and 5-HT_2C_ receptors play an important role in mediating the influence of serotonin in drug abuse and in the rewarding properties of drugs (Bubar and Cunningham, 2006, 2008; Craige and Unterwald, 2013; Howell and Cunningham, 2015). Because of the regional distribution of those receptors in the brain, they exert opposing effects on drug abuse through dopamine-dependent mechanisms (Manvich et al., 2012a,b; Murnane et al., 2013; Berro et al., 2017). Particularly, activation of 5-HT_2C_ and blockade of 5-HT_2A_ receptors have been proposed to decrease dopamine levels in the nucleus accumbens (NAcc) (Howell and Cunningham, 2015). Of note, the development and induction of CPP are dependent on drug-induced increases in NAcc dopamine levels (Koob and Bloom, 1988).

Although DMT is a 5HT_2A/2C_ receptor agonist, studies have shown that it has higher affinity for 5-HT_2A_ compared to 5-HT_2C_ receptors (Glennon et al., 2000). Thus, we propose that DMT-induced activation of 5-HT_2_ receptors would have distinct rewarding properties depending on the dose being administered. At low to intermediate doses, DMT would bind preferentially at 5-HT_2A_ receptors, which would then mediate its psychoactive properties. By activating 5-HT_2A_ receptors, which are highly expressed in dopaminergic neurons in the ventral tegmental area (VTA) (Howell and Cunningham, 2015), DMT would increase dopamine levels in the NAcc and exert rewarding effects. This mechanism would be responsible for the CPP induced by Aya at an intermediate dose in the present study. However, with increased dosage, DMT would then bind non-specifically at both 5-HT_2A_ and 5-HT_2C_ receptors. Activation of 5-HT_2C_ receptors, which are highly expressed in GABAergic interneurons within the VTA (Howell and Cunningham, 2015), would antagonize the effects of 5-HT_2A_ receptor activation on NAcc dopamine neurochemistry and prevent the development of CPP to Aya at higher doses, as observed in the present study.

Based on the hypothesis that higher doses of DMT would exert higher 5-HT_2C_ receptor activation, EPv would also be expected to induce CPP at intermediate, but not high, doses. In the present study, however, EPv did not induce CPP at any of the doses tested. The lack of effects observed for EPv could be due to the administration route used for EPv in the present study. While Aya and EBc were administered orally, EPv was administered i.p. due to expected DMT breakdown by MAO-A in the gastrointestinal tract. Of note, previous studies in humans have suggested that β-carboline-induced MAO inhibition is mainly peripheral and short-lived, and only allows around 15% of the DMT to reach systemic circulation (Riba, 2003; Domínguez-Clavé et al., 2016). Thus, with oral administration of Aya, the proportion of DMT that reaches the brain would be much lower than that of i.p. EPv, even though the DMT concentration in both extracts was the same across doses. Based on this hypothesis, i.p. EPv would be expected to induce CPP at lower doses than those used in the present study, with the doses of EPv used herein being high enough to exert 5-HT_2C_ agonist action, and thereby block DMT-induced CPP mediated by 5-HT_2A_ activation. In fact, although not statistically significant, increased dosage of EPv seemed to have a trend in inducing conditioned place avoidance under our experimental conditions (*p* = 0.1), which corroborates a higher 5-HT_2C_-mediated decrease in NAcc dopamine levels induced by higher doses of i.p. EPv.

Conditioning with EBc was not effective in inducing CPP under our experimental conditions, suggesting that its effects at 5-HT_2A/2C_ receptors at the doses tested in the present study were not meaningful. EBc also had no effects on the development of Eth-induced CPP. Aya, on the other hand, decreased CPP to Eth when administered before conditioning sessions. Interestingly, pretreatment with EPv also had no effects on Eth-induced CPP. Thus, our findings suggest that Aya-induced modulation of the development of Eth reward is dependent on the presence of both EBc and EPv, and seems to be a 5-HT_2A/2C_, rather than 5-HT_2C_, receptor-dependent mechanism. Ishiguro et al. (2016) have previously shown that the reduction of serotonergic neurons induced by chronic prenatal Eth exposure was alleviated by concomitant administration of a 5-HT_2A/2C_ receptor agonist. In this scenario, our findings suggest that Aya-mediated activation of 5-HT_2A/2C_ receptors blocked the development of Eth-induced CPP by preventing Eth-induced changes in serotonergic neurotransmission. An optimum ratio of 5-HT_2A/2C_ receptor activation seems to be necessary in order to exert those effects, because higher DMT doses (EPv) did not prevent Eth-induced CPP. Importantly, because all three treatments were capable of decreasing the expression of Eth-induced CPP, one cannot rule out the possibility that Aya might also be exerting rewarding effects and affecting the development of Eth-induced CPP via other chemicals that are not present in the plant extracts but are present in the Aya concoction.

Our findings also show that chronic treatment with Aya after Eth conditioning blocked the subsequent expression of CPP to Eth. Thus, Aya-mediated activation of 5-HT_2A/2C_ receptors also seems to modulate previously established Eth reward. This effect seems to be mediated not only by the presence of DMT, but also by the presence of β-carbolines in the Aya preparation. When administered in the previously Eth-associated environment, a high dose of EBc, as well as both doses of EPv, also blocked the expression of CPP to Eth. Therefore, although 5-HT_2A/2C_ activation seems to be sufficient and more effective at blocking the expression of CPP to Eth, β-carboline-mediated mechanisms also appear to modulate behavioral responses induced by Eth-environment conditioning.

As previously mentioned, β-carbolines have been proposed to exert psychoactive effects through 5-HT_2A/2C_ receptors. However, those mechanisms do not seem to account for the results obtained in the present study because EBc did not induce CPP alone or exert effects on the development of Eth-induced CPP. Instead, we hypothesize that other β-carboline-mediated mechanisms are responsible for the effects of EBc on the expression of Eth-induced CPP. β-carbolines have been shown to exert several effects in brain neurotransmission, not only through MAO-A inhibition and serotonin receptor binding, but also through dual specificity tyrosine-dependent kinase 1A (DYRK1A) inhibition, dopamine transporter (DAT) inhibition, and imidazoline I_2_ receptor binding (Brierley and Davidson, 2012). Thus, although the mechanisms underlying EBc-induced blockade of Eth-induced CPP remain unknown, this is the first study to show that EBc alone could exert therapeutic potential for the treatment of ethanol abuse without exerting addictive properties.

Finally, the effects observed with Aya treatment were context-dependent: when administered in the previously Eth-associated compartment, its effects were observed for both doses tested, while only the intermediate dose blocked the expression of Eth-induced CPP when Aya was administered in the Sal-paired compartment. Therefore, Aya’s ability to induce rewarding effects predicted its ability to block the expression of CPP to Eth when Aya was administered in a neutral (Sal-paired) environment. By exerting rewarding properties when administered in a neutral compartment, a rewarding dose of Aya would modify the incentive salience of the CPP apparatus (Valyear et al., 2017), and animals would no longer express CPP to Eth during the post-treatment test.

When given in the Sal-paired compartment, EPv also blocked the expression of Eth-induced CPP at all doses tested, suggesting that activation of 5-HT_2A/2C_ receptors mediates those effects of Aya. Corroborating our previous hypothesis, high doses of i.p. EPv would be expected to induce higher activation of 5-HT_2C_ receptors, which would ultimately decrease NAcc dopamine levels and block the expression of CPP to Eth (Pina and Cunningham, 2014). However, similarly to Aya, EBc blocked the expression of Eth-induced CPP when administered at an intermediate dose in the Sal-paired compartment. Thus, our findings suggest that increasing the dose of EBc in the Aya mixture seems to counteract some of the effects of EPv. Because this effect was only observed in an experimental condition associated with low brain dopamine levels (presentation to a neutral, previously habituated environment), EBc seems to modulate Eth-induced CPP through mechanism that are dependent on dopamine availability.

Finally, a parameter that could be influencing the effects observed herein is locomotor activity. Treatment with Aya and its components could have affected locomotor activity in animals conditioned to Eth, and therefore decreased time spent in the Eth-associated compartment during the post-treatment test by increasing locomotor activity and exploration (Experiments 3–5). When looking into locomotor activity data in the present study, we observed that during the post-conditioning test, animals showed significantly higher levels of CPP score and locomotor activity score (difference between the distance traveled in the Eth- and Sal-paired compartments) compared to the pre-conditioning test (Experiments 3, data not shown). Those data suggest that animals spent more time and explored more the Eth-paired compartment compared to the Sal-paired compartment. During the post-treatment test, however, no statistically significant differences were observed for locomotor activity scores between groups (Experiments 3, data not shown). Animals no longer showed a difference between the distance traveled in the Sal- vs. Eth-paired compartments, suggesting a habituation factor resulting in lower levels of exploratory behavior. Those results corroborate previous findings from our laboratory showing that the same Aya extract used in the present study did not affect baseline locomotion or induce locomotor sensitization in mice, and instead decreased Eth-induced hyperlocomotion and locomotor sensitization (Oliveira-Lima et al., 2015). Importantly, the results obtained for locomotor activity did not correlate to the results obtained for time score, as animals in the Vehicle control group, for instance, still expressed CPP do Eth during the post-conditioning test, an effect that was blocked by treatment with Aya. Thus, analysis of locomotor activity data does not support an influence of this parameter on the expression of CPP to Eth.

In summary, although we have not directly investigated the molecular mechanisms underlying the present findings, our results suggest that the expression of the Aya reward is dependent on a specific 5-HT_2A/2C_ occupancy ratio that can be modulated by the quantity of EBc and EPv present in the Aya batch and by the route of administration. Of clinical importance, we propose that a proper 5-HT_2A/2C_ occupancy could engender therapeutic efficacy without exerting abuse liability. By showing that both EBc and EPv exerted therapeutic effects alone, our findings also indicate that the combination of EBc and EPv in the form of Aya beverage seems to have unique therapeutic utility. Importantly, under our experimental conditions, the treatment environment influenced the therapeutic effects of Aya and EBc, but not EPv, which could be relevant in the context of clinical and/or religious/ritual uses of Aya. The present study further emphasizes the complexity of the Aya formulation and its effects, and highlights the need for further investigations in the area. Future studies investigating the effects of varying EBc/EPv ratios on the therapeutic properties of Aya are needed in order to ensure the safety and best therapeutic strategy in using this beverage for the treatment of ethanol abuse.

## Author Contributions

AO-L, LB, and EM were responsible for the study concept and design. EC-P, YS, EM-J, HR, NK, ML-S, RS, TB-S, LS, and JC contributed to the acquisition of data. EC-P, PB, LB, and EM assisted with data analysis and interpretation of findings. EG-P, PB, LB, and EM drafted the manuscript. All authors provided critical revision of the manuscript for important intellectual content and approved final version for publication. All authors agree to be accountable for all aspects of the work in ensuring that questions related to the accuracy or integrity of any part of the work are appropriately investigated and resolved.

## Conflict of Interest Statement

The authors declare that the research was conducted in the absence of any commercial or financial relationships that could be construed as a potential conflict of interest.

## Abbreviations

Aya: ayahuasca
EBc: extract of *Banisteriopsis caapi*
EPv: extract of *Psychotria viridis*
Eth: ethanol
